# Supplementary material for: A Global Screen for Assembly State Changes of the Mitotic Proteome by SEC-SWATH-MS
Source: Cell Syst. 2020 Feb 26;10(2):133–155.e6. doi: 10.1016/j.cels.2020.01.001 (PMC7042714; doi:10.1016/j.cels.2020.01.001)

Q63ZY3 | KANK2_HUMAN | KANK2 ANKRD25 KIAA1518 MXRA3 SIP
Monomer MW [kDa]: 91.174 Monomer expected elution fraction: 41

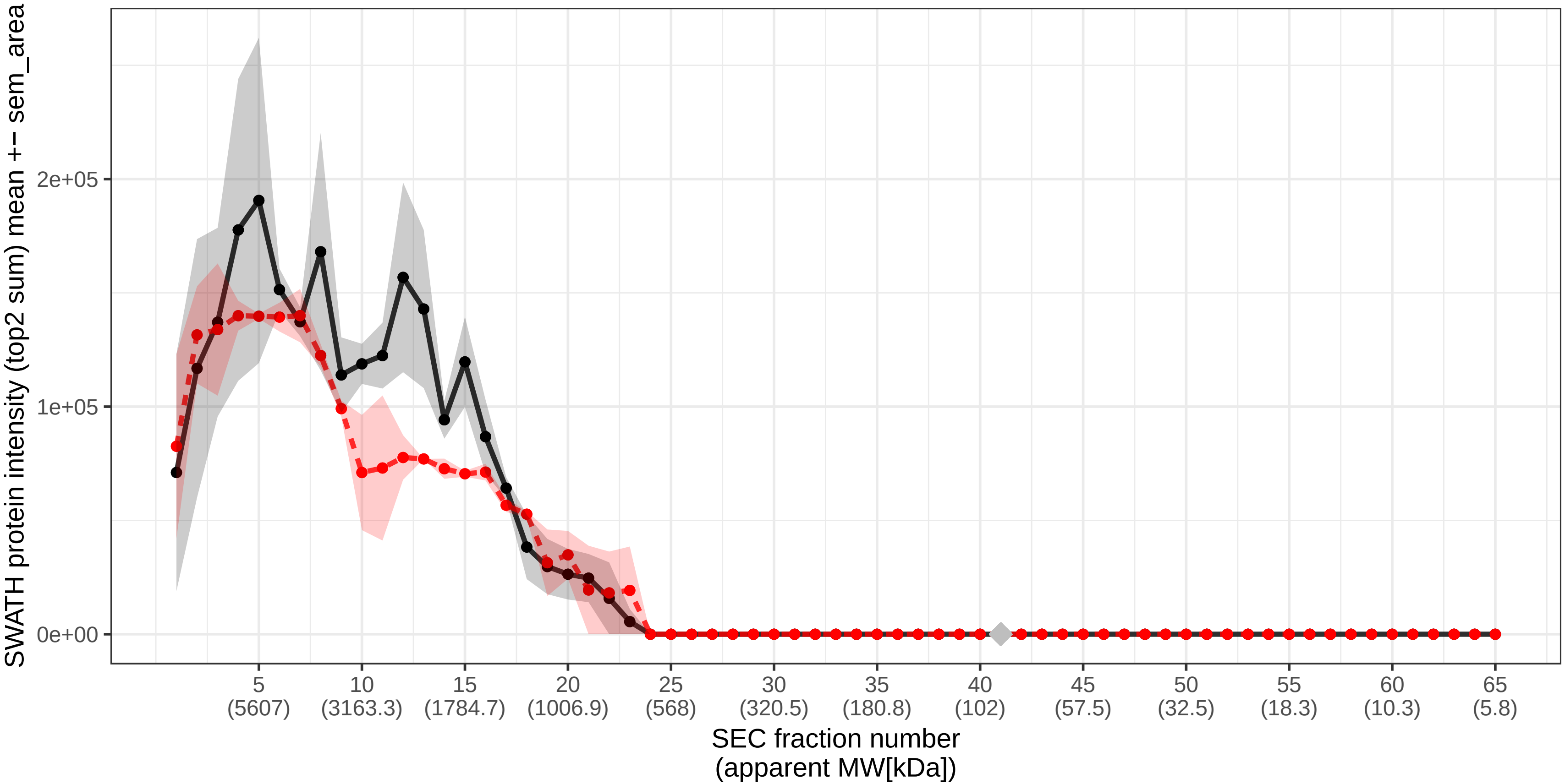

Supplement: Data S1. SEC-SWATH-MS Protein Chromatograms, Related to Figure 1 [file mmc6.zip › SECchrom_Q63ZY3_KANK2_HUMAN_KANK2_ANKRD25_KIAA1518_MXRA3_SI.pdf]
